# Brain Basis of Psychopathy in Criminal Offenders and General Population

**DOI:** 10.1093/cercor/bhab072

**Published:** 2021-04-09

**Authors:** Lauri Nummenmaa, Lasse Lukkarinen, Lihua Sun, Vesa Putkinen, Kerttu Seppälä, Tomi Karjalainen, Henry K Karlsson, Matthew Hudson, Niina Venetjoki, Marja Salomaa, Päivi Rautio, Jussi Hirvonen, Hannu Lauerma, Jari Tiihonen

**Affiliations:** 1 Turku PET Centre, and Turku University Hospital, University of Turku, Turku 20520, Finland; 2 Department of Psychology, University of Turku, 20014, Finland; 3 Psychiatric Hospital for Prisoners, Health Care Services for Prisoners, Turku FI-20251, Finland; 4 Turku Prison Outpatient Clinic, Health Care Services for Prisoners, Turku, FI-20251, Finland; 5 Department of Radiology, Turku University and Turku University Hospital, Turku, Finland, Turku 20520, Finland; 6 Department of Clinical Neuroscience, Karolinska Institutet and Center for Psychiatry Research, Stockholm City Council, Stockholm SE-11364, Sweden; 7 Department of Forensic Psychiatry, University of Eastern Finland, Kuopio 70240, Finland

**Keywords:** empathy, fMRI, psychopathy, VBM, violence

## Abstract

Psychopathy is characterized by persistent antisocial behavior, impaired empathy, and egotistical traits. These traits vary also in normally functioning individuals. Here, we tested whether such antisocial personalities are associated with similar structural and neural alterations as those observed in criminal psychopathy. Subjects were 100 non-convicted well-functioning individuals, 19 violent male offenders, and 19 matched controls. Subjects underwent T1-weighted magnetic resonance imaging and viewed movie clips with varying violent content during functional magnetic resonance imaging. Psychopathic traits were evaluated with Levenson Self-Report Psychopathy Scale (controls) and Psychopathy Checklist-Revised (offenders). Psychopathic offenders had lower gray matter density (GMD) in orbitofrontal cortex and anterior insula. In the community sample, affective psychopathy traits were associated with lower GMD in the same areas. Viewing violence increased brain activity in periaqueductal grey matter, thalamus, somatosensory, premotor, and temporal cortices. Psychopathic offenders had increased responses to violence in thalamus and orbitofrontal, insular, and cingulate cortices. In the community sample, impulsivity-related psychopathy traits were positively associated with violence-elicited responses in similar areas. We conclude that brain characteristics underlying psychopathic spectrum in violent psychopathy are related to those observed in well-functioning individuals with asocial personality features.

## Introduction

Psychopathy is a personality disorder characterized by persistent antisocial behavior, impaired empathy and remorse, as well as bold, disinhibited, and egotistical traits (Cooke and Michie 2001). In clinical and forensic settings, psychopathy predicts criminal behavior and violence (Salekin et al. 1997). For example, whereas prevalence of psychopathy is ~1% in normal population, it is ~20% in incarcerated offenders (Hare 1991; in Finland 16.4%, Juriloo et al. 2014). The pervasive nature of both behavioral and emotional symptoms suggest that psychopathy might have organic basis, and multiple studies have found that psychopathic offenders have atrophy in frontal cortex and in limbic regions including insula and amygdala (Muller et al. 2008; Tiihonen et al. 2008; Yang et al. 2009; Ermer et al. 2012). These structural impairments are coupled with abnormal function of the limbic system, and psychopathic subjects show less affect-related activity in amygdala and hippocampus, striatum and cingulate cortices while viewing emotional facial expressions, and stronger activation of frontal cortical regions (Kiehl et al. 2001; Dolan and Fullam 2009).

When viewing empathy-eliciting scenes, psychopathic individuals show significantly reduced frontocortical brain activity compared with healthy controls, consistent with their lowered care motivation (Decety et al. 2013; Meffert et al. 2013). The weakened limbic outputs combined with dysfunction in executive frontal cortical and social decision-making systems could thus predispose psychopaths to violent and antisocial behavior. Finally, conduct disorder (CD) is predictive of adult psychopathy (Burke et al. 2007). Similarly to adult psychopaths, adolescents with CD have reduced gray matter volume in amygdala and insula (Fairchild et al. 2011). Some studies have found that participants with CD show increased amygdala response to neutral but not angry faces (Passamonti et al. 2010), whereas another study has found that adolescents with conduct problems and low callous-unemotional traits show heightened amygdala responses to briefly presented fearful faces (Viding et al. 2012).

Most people never commit a crime or any sort of violent attack, yet there is considerable variation in everyday antisocial and mildly delinquent behavior. For example, ~60% of people lie during casual conversations (Feldman et al. 2002), in EU 40–60% of drivers regularly exceed speeding limits (EuropeanCommission 2020), and ~10% of the US population have used illicit drugs (NIDA 2013). It has thus been proposed that psychopathy is not a categorical designation similarly as, for example, clinical diagnoses. Instead, it may be better to view psychopathy as a constellation of continuous, lower-order antisocial, and aversive personality dimensions that vary in the non-incarcerated population with normal range of social functioning (Levenson et al. 1995; Miller et al. 2008). This hypothesis is supported by data from community samples where high scores on psychopathic traits are associated with antisocial behavior such as aggression and racism (Hodson et al. 2009; Jones and Paulhus 2010). This variation is also reflected in cortical structure: psychopathic traits in general populations are associated with decreased gray matter density (GMD) in striatum and amygdala (Vieira et al. 2015), whereas some studies have found regional increased cortical density in frontal cortical regions (Lasko et al. 2019). In healthy subjects, high levels of psychopathic traits are associated with heightened brain responses to facial expressions in amygdala and frontal cortex (Gordon et al. 2004). However, interpretation of these studies is difficult due to compromised sample sizes and typically lack of direct comparison with criminal psychopathic offenders.

### The Current Study

Forensic imaging studies on psychopathy are complicated by clinical confounding factors and substance use history, even though the large psychological differences between psychopaths and healthy controls makes them well powered (Koenigs et al. 2011). Studies focusing on variation in psychopathic traits in the general population have the advantage of better control over comorbid psychiatric illnesses and substance use, yet the restricted variation in psychopathic traits and corresponding self-reported trait measures compromises the statistical power, which has been identified as a major problem in neuroimaging studies (Cremers et al. 2017; Poldrack et al. 2017). Finally, functional neuroimaging experiments on psychopathy have measured brain responses to isolated features such as static faces or words that are not representative of the dynamic and ever-changing real world, and there is prima facie doubt regarding the generalizability of this approach to real-world social behavior (Adolphs et al. 2016). Here, we tested whether psychopathic traits in a large well-functioning community sample (*n* = 100) are associated with 1) cortical density and 2) brain activity when viewing highly naturalistic violent episodes, and 3) whether these effects match with corresponding structural and functional alterations in convicted violent offenders (*n* = 19) with psychopathic traits versus matched healthy controls (*n* = 19). We show that in the community sample, the degree of psychopathic traits is associated with lowered fronto-limbic structural integrity and elevated frontal and insular hemodynamic brain activity while seeing violence, and that these alterations are similar with those observed in the forensic sample.

## Materials and Methods

All subjects gave an informed, written consent and were compensated for their participation. The ethics board of the Hospital District of Southwest Finland approved the protocol and the study was conducted in accordance with the Declaration of Helsinki. A total of 100 non-convicted volunteers drawn from the community volunteer pool participated in the study (51 females, mean age 31 years, range 20–57 years; see Table 1). The exclusion criteria included a history of neurological or psychiatric disorders, alcohol and substance abuse, current use of medication affecting the central nervous system, medical conditions precluding participation, and the standard magnetic resonance imaging (MRI) exclusion criteria. Four additional subjects were scanned but 2 were excluded from analyses because their MRIs revealed gross brain abnormalities, and 2 others due to malfunctioning gradient coil resulting in unusable data. In a separate forensic imaging experiment, 19 convicted violent male offenders with high psychopathic traits and 19 age and sex-matched control subjects (different from those described previously) were studied (Table 1). See Supplementary Figure S1 for the distributions of the primary and secondary psychopathy scores. All offenders were inmates from the Turku Prison currently serving a sentence for either murder (*n* = 5), manslaughter (*n* = 5), attempted manslaughter (*n* = 3) or grievous bodily harm (*n* = 6). Mean recidivism rate was 2.4 times after first conviction. All were screened for illicit drug use both in the screening and on the day of the MRI scan. Information on medication and psychiatric diagnoses of the forensic subjects is presented in Supplementary Tables S1 and S2. Forensic subjects were escorted to the hospital imaging site by 2 prison guards who monitored the whole study protocol.

**Table 1:** Subject characteristics with frequencies and means with standard deviations in parenthesis

	Community sample	Convicted offenders	Matched controls
*n* (Males)	49	19	19
*n* (Females)	51	0	0
Age	31.14 (9.31)	31.16 (6.49)	28.53 (7.69)
BMI	23.86 (2.61)	28.05 (3.90)	25.10 (2.09)
PCL-R	—	26.47 (6.24)	—
LSRP primary psychopathy	23.42 (4.07)	—	21.94 (3.26)
LSRP secondary psychopathy	15.99 (3.18)	—	13.56 (3.00)

**Figure 1:**
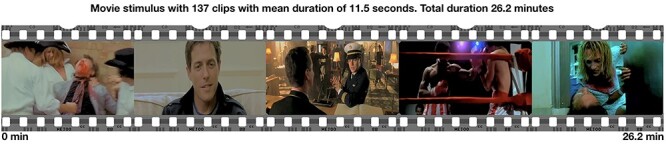
Experimental design and sample stimuli. The subjects viewed a compilation of 137 movie clips with variable violent and nonviolent content.

Psychopathy in the convicted offenders was assessed with the Hare Psychopathy Checklist-Revised (PCL; Hare 1991), based on semi-structured interview by an experienced forensic psychologist/psychiatrist and review of collateral information. The PCL-R measures 2 dimensions of psychopathic traits: primary psychopathy involving inclination to lie, lack of remorse, and callousness, and secondary psychopathy involving impulsivity, short temper, and low tolerance for frustration. Psychopathic traits in the non-convicted population were measured with the Levenson Self-Report Psychopathy Scale (LSRP; Levenson et al*.* 1995). This self-report instrument is based on the 2-factor (primary/secondary) conceptualization of the PCL. One subject had incomplete items in the secondary psychopathy scale items and was left out from the corresponding analyses.

### Experimental Design for functional magnetic resonance imaging

To map brain responses to seeing violence, we used our previously validated socioemotional “localizer” paradigm that allows reliable mapping of various social and emotional functions (Lahnakoski et al. 2012; Karjalainen et al. 2017, 2019). Briefly, the subjects viewed a medley of 137 movie clips (mean duration 11.5 s; total duration 26.2 min) that have been curated to contain large variability of social and emotional content (Fig. 1). All videos were extracted from mainstream Hollywood movies with audio track in English. Because this task is designed to map neural processing of naturalistic socioemotional events, the clips are not deliberately matched with respect to, for example, human motion or optic flow. The videos were presented in fixed order across the subjects without breaks. Subjects were instructed to view the movies similarly as if they were viewing a movie at a cinema or at home, that is, no specific task was assigned. Visual stimuli were presented with NordicNeuroLab VisualSystem binocular display. Sound was conveyed with Sensimetrics S14 insert earphones. Stimulation was controlled with Presentation software. Before the functional run, sound intensity was adjusted for each subject so that it could be heard over the gradient noise. Six independent raters evaluated the occurrence of violence in the movie clips with 4-s temporal accuracy. These ratings were subsequently averaged and used as regressors in the functional magnetic resonance imaging (fMRI) data analysis (see below). Due to time constraints (the offenders also participated in another study on the same visit), the task used for the offender sample was slightly trimmed in duration (12 min). Our previous work however shows that the task is robust to this kind of variations in the stimulus material selection (Lahnakoski et al*.* 2012; Karjalainen et al*.* 2017, 2019).

### MRI Data Acquisition and Preprocessing

The MRI data were acquired using a Phillips Ingenuity TF PET/MR 3T whole-body scanner. High-resolution (1 mm^3^) structural images were obtained with a T1-weighted sequence (time repetition [TR] 9.8 ms, time echo [TE] 4.6 ms, flip angle 7°, 250-mm FOV, and 256 × 256 reconstruction matrix). A radiologist screened the T1 images for structural abnormalities. A total of 407 functional volumes were acquired with a T2^*^-weighted echo-planar imaging sequence (TR = 2600 ms, TE = 30 ms, 75° flip angle, 240 mm FOV, 80 × 80 reconstruction matrix, 62.5 kHz bandwidth, 3.0-mm slice thickness, and 45 interleaved slices acquired in ascending order without gaps). One functional run with 476 functional images was acquired (275 volumes for the forensic sample and their controls). MRI data were preprocessed using fMRIPprep 1.3.0.2 (Esteban et al. 2019). The following preprocessing steps were performed on the anatomical T1-weighted (T1w) reference image: correction for intensity non-uniformity, skull-stripping, brain surface reconstruction, and spatial normalization to the ICBM 152 Nonlinear Asymmetrical template version 2009c using nonlinear registration with antsRegistration (ANTs 2.2.0) and brain tissue segmentation. The following preprocessing steps were performed on the functional data: co-registration to the T1 reference image, slice-time correction, spatial smoothing with a 6-mm Gaussian kernel, automatic removal of motion artifacts using ICA-AROMA (Pruim et al. 2015), and resampling to the MNI152NLin2009cAsym standard space.

**Figure 2:**
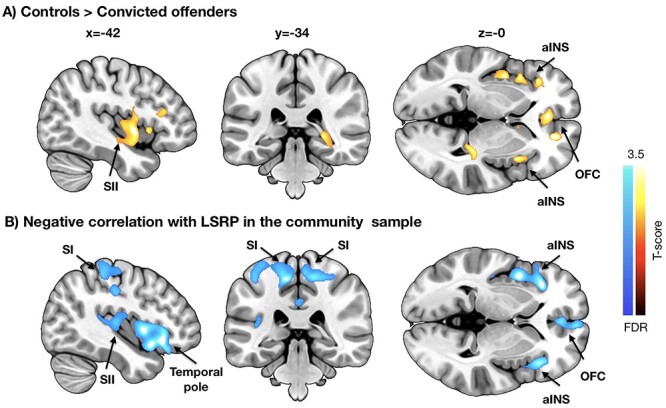
Results from the voxel-based morphometry analyses. (*A*) Brain regions where convicted offenders had significant atrophy in comparison with control subjects. (*B*) Brain regions where primary psychopathy scores were associated with decreased GMD, *P* < 0.01 FDR corrected. aINS = anterior insula, OFC = orbitofrontal cortex, SI = primary somatosensory cortex, SII = secondary somatosensory cortex. .

### Voxel-Based Morphometry

Voxel-based morphometry (VBM) was done with SPM12 (Wellcome Trust Center for Imaging, London, United Kingdom, http://www.fil.ion.ucl.ac.uk/spm), which enables automated spatial normalization, tissue classification and radio-frequency bias correction to be combined with the segmentation step. Cutoff of spatial normalization was 25 mm and medium affine regularization 0.01 was used. Following normalization and segmentation into grey and white matter, a modulation step was incorporated to take into account volume changes caused by spatial normalization. Importantly, the modulation step corrects for the differences in total brain size across subjects. Finally, the segmented, normalized, and modulated GMD/WMD images were smoothed using a Gaussian kernel of 8-mm full width at half maximum. Normalized and smoothed images were analyzed with general linear model (GLM) where they were predicted with primary and secondary psychopathy scores, while age and sex (for the sample of 100 non-convicted volunteers only; the forensic sample and their controls were all males) were entered as nuisance covariates. Primary statistical threshold was set at *P* < 0.01, FDR corrected at cluster level.

### fMRI Data Analysis

The fMRI data were analyzed in SPM12. To reveal brain regions activated by violence, a subjectwise (first-level) GLM was fitted to the data, where the blood oxygen level–dependent (BOLD) signals were predicted with the violence regressors convolved with the canonical hemodynamic response function. To identify the brain regions showing enhanced brain activity consistently across the subjects, the individual beta images for the violence regressor were the entered to a second-level analysis. The effects of primary and secondary psychopathy were finally assessed by adding them as covariates into the second-level models. Primary statistical threshold was set at *P* < 0.05, False Discovery Rate (FDR) corrected at cluster level.

### Connectivity Analyses

To test whether interregional coupling during movie viewing varies across the psychopathic offenders and healthy controls (and as a function of psychopathic traits in the community sample), we performed connectivity analyses. We first extracted regional time courses from the detrended BOLD time series from a priori regions of interest involved in emotional processing including amygdala, insula, frontal pole, and thalamus (Saarimäki et al. 2016, 2018). These subjectwise time series were then used as regressors in first level models, and the resulting contrast images were analyzed using GLM for population-level inference similarly as in the VBM and BOLD-GLM analyses.

## Results

### VBM

Voxel-based morphometry (Fig. 2) revealed that offenders with psychopathic traits had significantly lowered GMD in anterior insula, orbitofrontal cortex (OFC) and secondary somatosensory cortex (SII), indicating atrophy in these regions. No opposite effects were found. In the community sample, negative association with primary LSRP scores (i.e., lower cortical density with higher psychopathy scores scores) was observed in the same regions. Additional effects were observed in primary somatosensory cortex (SI), paracentral lobule and midcingulate cortex. No regions showed significant positive associations with primary psychopathy even with a more lenient threshold (*P* < 0.05 FDR corrected), and neither positive nor negative associations were found with secondary psychopathy. Analysis of the WM segments revealed only lowered density in the cerebellum and around the lingual gyrus in the psychopathic group. No opposite effects were found. In the community sample, there were no positive or negative associations with neither primary nor secondary psychopathy scores.

**Figure 3:**
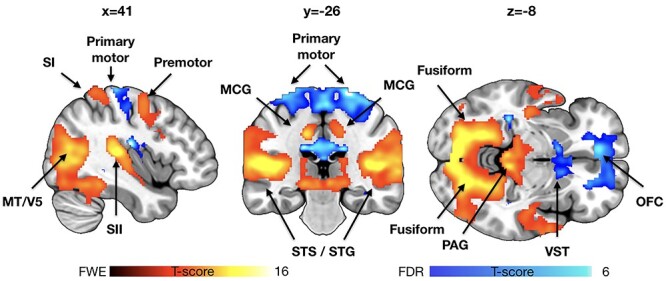
Brain regions showing increased (hot colors, 0.05 familywise error (FWE) corrected for visualization) and decreased (hot colors, *P* < 0.05 FDR corrected for visualization) activity as a function of violence seen in the movies. Data for the community sample are shown for reference. MCG = middle cingulate gyrys, STG = superior temporal gyrus, STS = superior temporal sulcus, and VST = ventral striatum.

### fMRI

Viewing violent scenes elicited widespread and strong brain activation (Fig. 3). The effects spanned limbic and paralimbic emotion circuits (thalamus, periaqueductal gray [PAG], and middle cingulate cortex) as well as the somatosensory cortices (SI–SII) and components of the frontoparietal network (inferior parietal and lateral premotor cortices). Significant activation clusters were also observed in visual (V1 and the MT–V5 complex) and auditory cortices, as well as in regions involved in face and social perception, such as the lateral fusiform gyrus and posterior polysensory areas of the superior temporal gyrus and sulcus. Deactivations were significantly weaker than activations, and were localized to primary motor cortex, OFC, and ventral striatum.

**Figure 4:**
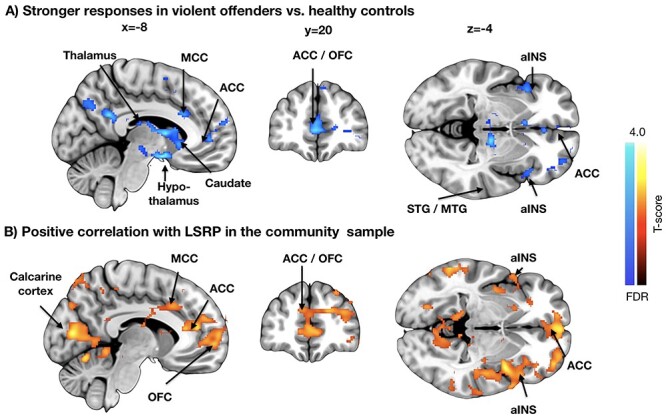
Brain regions whose responses to seen violence were stronger on convicted offenders versus controls (*A*) and positively associated with secondary psychopathy scores in the community sample (*B*). The data are thresholded at *P* < 0.05, FDR corrected. MCC = middle cingulate cortex.

We then compared how the brain responses to seeing violence in the psychopathic offenders differ from those of healthy controls (Fig. 4). In the psychopathic offenders the violent scenes provoked heightened activation in OFC, bilateral insula, anterior and middle cingulate cortices, thalamus, and superior and middle temporal polysensory regions. Next, we assessed whether the degree of psychopathic traits associates with brain responses to violent scenes in the well-functioning community sample, and found that the effects were comparable with those observed in the convicted offenders. The strongest positive associations were observed for the secondary psychopathy scores, which were positively associated with violence-dependent BOLD responses in orbitofrontal and anterior insular cortices and along the whole anterior–posterior axis of the cingulate cortex, and SI, and also in the visual cortices. Effects for primary psychopathy were weaker but overlapped with those of secondary psychopathy in the orbital and frontal areas.

**Figure 5:**
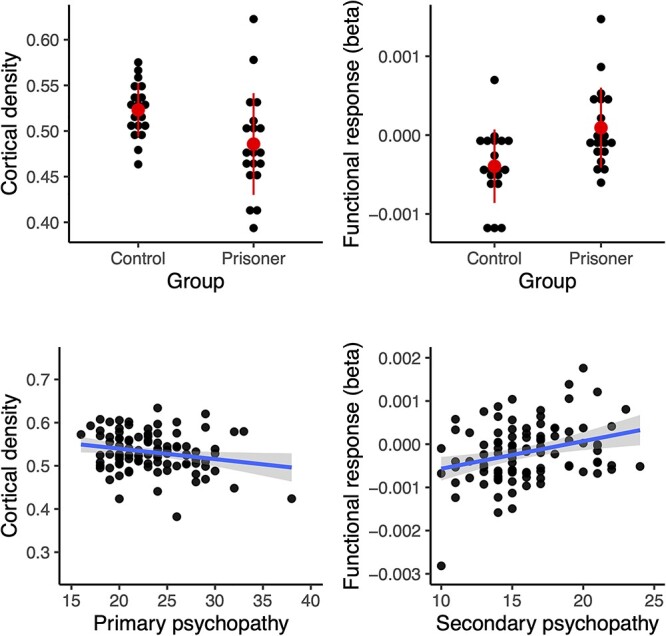
Orbitofrontal cortical density (left; expressed as probability of a voxel belonging to gray matter) and functional responses (right) in the prisoners and controls (top) and in the community sample (bottom). Error bars in the dotplots show ±2 standard deviation, the scatterplots show LS-regression lines with 95% confidence interval.

Finally, to confirm the consistency of psychopathy-dependent orbitofrontal alterations (that were consistently observed in all the analyses) and to address the direction of the associations between psychopathy and brain structure and function, we generated a 6-mm spherical region of interest (ROI) in OFC. The ROI was centered around a peak voxel (*x* = 1, *y* = 46, and *z* = 2) showing strong positive association between psychopathic traits and violence-elicited brain responses in the community sample. Next, we extracted regional cortical density (for VBM data) and beta (for fMRI) values in this ROI for both the community sample and the prisoners and their respective controls. This analysis (Fig. 5) showed that in OFC, psychopathic offenders had increased atrophy (i.e., lowered cortical density; *t* = 2.59 and *P* = 0.015) and increased responses to seen violence comparison with healthy controls (i.e., betas, *t* = 3.08 and *P* = 0.003). The results were comparable in the community sample—in the OFC primary psychopathy was negatively associated with cortical density (*r* = 0.22 and *P* = 0.028), while secondary psychopathy was positively associated with the functional responses to seen violence (*r* = 0.30 and *P* = 0.002). Note that this analysis could be considered “circular” (Kriegeskorte et al. 2009) for the functional responses in the community sample that was used for ROI definition, but not in the 3 other conditions (structural effects in community sample, functional and structural effects in the prisoners, and controls). This analysis thus suggests that there are consistent psychopathy-related structural and functional alterations in the orbitofrontal region.

### Connectivity Analyses

**Figure 6:**
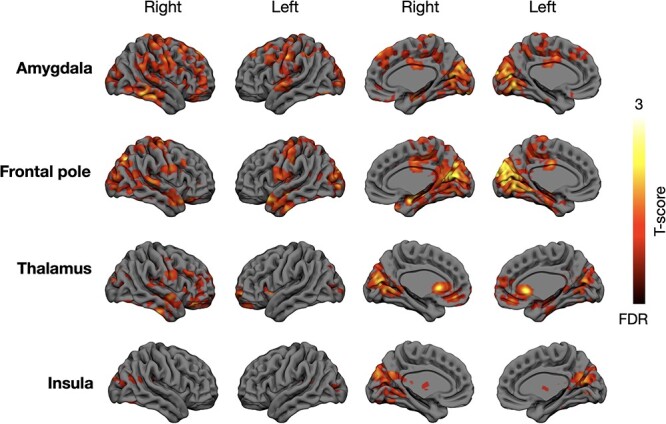
Brain regions whose connectivity with amygdala, frontal pole, thalamus, and insula was lowered in the convicted offenders versus controls. The data are thresholded at *P* < 0.05, FDR corrected at cluster level.

The connectivity analyses revealed consistent hypoconnectivity in the psychopathic offenders (Fig. 6). All the tested regions showed lowered connectivity with cuneus and calcarine cortex. The largest differences were observed for amygdala and frontal pole, whose hypoconnectivity spanned inferior temporal cortex and supramarginal gyrus, S1 and S2, cingulate and lateral and medial frontal cortex as well as caudate and putamen. For thalamus, significant hypoconnectivity was observed with insula, caudate and lateral frontal cortex. Altered connectivity from insula was primarily restricted to cuneus and calcarine cortex. For the community sample, the results did not clearly parallel with those obtained in the forensic sample. Only connectivity from amygdala, thalamus and frontal to calcarine cortex was negatively associated with primary (frontal pole) and secondary (amygdala and thalamus) psychopathy scores, thus resembling the convicted offenders. Largest discrepancies were observed for thalamus and insula, whose connectivity with medial temporal, cortical midline, and lateral and medial frontal cortices were associated positively with the primary psychopathy scores.

## Discussion

Our main findings were that 1) psychopathic traits among healthy individuals are associated with compromised cerebral structural integrity and amplified functional responses to seeing highly naturalistic violence and that 2) these alterations resemble the differences observed between incarcerated violent psychopathic offenders and healthy controls. Primary (affective) psychopathic traits were associated with compromised gray matter integrity in the medial frontal, insular, and somatosensory cortices, whereas secondary (impulse control related) psychopathic traits were associated with increased functional responses in these areas when viewing violent videos. Together these data provide structural and functional neurobiological evidence for the similar neural basis of criminal psychopathy and antisocial yet psychologically well-functioning personalities.

### Psychopathic Traits Are Associated with Cerebral Integrity

The violent offenders had lowered GMD in OFC and anterior insulae. In the community sample, primary psychopathy scores were associated with lowered cortical integrity in the same areas. This effect was also specific to grey matter, and we found no evidence of aberrant white matter density as a function of psychopathy except in the cerebellum of the convicted sample. These results bear striking resemblance to prior VBM studies on criminal psychopaths. These studies have found significant psychopathy-dependent atrophy in OFC/ACC (anterior cingulate cortex) and insula (Tiihonen et al. 2008; Ermer et al. 2012), suggesting that similar neural alterations underlie criminal psychopathy and variation in everyday antisocial behavior in psychologically well-functioning individuals.

Frontal lobe dysfunction and poor executive functions are hallmarks of disorders involving impulsivity (Morgan and Lilienfeld 2000; Yang and Raine 2009). Numerous studies have reported lowered glucose metabolism in frontal and temporal lobes in violent psychiatric patients and murderers (Raine et al. 1994, 1997, 1998; Volkow et al. 1995; George et al. 2004), and the present data align well with these observations by demonstrating psychopathy-related alterations in the same brain regions in a healthy sample. Previous studies have also found hippocampal and amygdala density reductions in psychopathic offenders (Ermer et al. 2012). Although these effects were not manifested in our data with the a priori statistical threshold, it is noteworthy that with slightly more liberal (yet multiple comparison corrected) threshold, primary psychopathy scores were also negatively associated with GMD in amygdala and hippocampus (*P* < 0.05, FDR corrected, data not shown).

Prior structural imaging studies have yielded mixed results concerning the cerebral alterations associated with psychopathy: Whereas many studies have found decreased frontocortical and limbic densities in psychopathy (Tiihonen et al. 2008; Ermer et al. 2012), some have found positive associations between frontal/OFC densities and secondary (factor 2) psychopathy (Korponay et al. 2017). These discrepancies could be attributed to a multitude of factors, such as definition of and the criteria used for psychopathy, whether incarcerated or typical populations with variation in psychopathy scores are studies, and subjects’ substance use history (Koenigs et al. 2011). The present study in turn clearly replicates the association between psychopathic traits and fronto-insular atrophy in both healthy volunteers (self-reports of psychopathy) and forensic sample (objective measures of psychopathy), providing strong evidence for this regionally specific cerebral alteration associated with psychopathy.

### Brain Responses to Seeing Violence

Viewing violence elicited consistent responses in widespread brain regions, including PAG that is a key part of the pain-inhibiting circuitry (Tracey and Mantyh 2007). PAG is also centrally involved in generating freezing responses to distal threats and reactive aggression during threats (Panksepp 1998; Gregg and Siegel 2001). Human fMRI studies have accordingly demonstrated that proximity of threats activates the PAG in humans (Mobbs et al. 2007). Significant activations were also found in thalamus, which likely modulates arousal levels during threatening events (Nummenmaa et al. 2008). Seeing violence also engaged the dorsal frontoparietal network involved in goal-directed attention shifts and sustained attention (Corbetta and Shulman 2002; Nummenmaa et al. 2017), likely resulting from focused attention on the survival-salient events (Vuilleumier 2005). Consistent activations were found in the lateral fusiform gyrus centrally involved in facial expression recognition (Calder and Young 2005), as well as posterior polysensory areas of the superior temporal sulci and gyri that integrate multitude of social signals and act as the centralized “hub” of social perception in humans (Nummenmaa and Calder 2009; Lahnakoski et al. 2012). Finally, seeing violence elicited significant activity in the motion-sensitive MT/V5 complex. This likely reflects the increased motion kinematics in the violent episodes, and post hoc analyses indeed revealed that violence in the movies was associated with increased perceived human motion (*r* = 0.17 and *P* < 0.05).

Altogether the coordinated activity of this network encodes the threat value of the environment, focuses attentional resources to the critical features and prepares the fight-or-flight response when needed. Remarkably, the increased activation of the somatosensory and premotor cortices was accompanied with significant deactivation in primary motor cortex and rostral ACC. Although speculative, it is possible that this distinction reflects the engagement of the violence inhibition mechanism, whereby seeing others’ distress leads to somatosensory (SI–SII) and premotor mirroring of others distress, leading to inhibitory activation at the motor cortex subsequently preventing engagement in violence. Previous fMRI studies on first-person simulated fighting have found that dorsal ACC responses are increased when executing violent actions, reflecting this region’s role in emotion regulation (Mathiak and Weber 2006).

### Psychopathic Traits Mediate Neural Responses to Violence

Violent psychopathic offenders showed increased hemodynamic responses to seeing violence in orbitofrontal and insular cortices, and also throughout the cingulate cortex. In the healthy community sample, secondary psychopathy scores were associated with increased hemodynamic responses in comparable brain regions, suggesting similarities between brain characteristics of criminal psychopathy and more benign forms of antisociality. It is also noteworthy that while structural alterations in the healthy sample were linked with primary psychopathic traits, the functional responses were associated with secondary psychopathic traits. It is likely that the latter reflects the contents of the naturalistic video stimulus which contained numerous episodes of impulsive violence, whose processing may not be modulated by callousness-related traits.

Prior functional imaging studies have found that psychopaths typically have increased rather than decreased functional responses to emotional stimuli, typically facial expression of fear (Kiehl et al. 2001; Dolan and Fullam 2009), yet to our knowledge our study is the first one to measure psychopathy-related neural responses to naturalistic violence scenes. One possible explanation for the amplified brain responses to violent scenes is that psychopathy involves impaired inhibitory control during affective provocation, which may lead to aggression during real-life encounters. It is noteworthy that OFC and insula–regions whose structural integrity was negatively associated with psychopathy–showed positive association between secondary psychopathy and BOLD responses to violence (Fig. 5). This might reflect a compensatory functional activity due to compromised cerebral integrity in these regions, and suggest a general role of the OFC in mediating antisocial behavior.

Both insula and SI are important for generating the bodily component of emotions (Damasio 1999; Nummenmaa et al. 2014, 2018). Furthermore, damage to the primary somatosensory cortex (Adolphs et al. 2000) or its inhibition with transcranial magnetic stimulation (Pourtois et al. 2004) impairs facial expression recognition. Psychopathic individuals show affective disengagement and dampened autonomic nervous system reactivity to a variety of emotional stimuli (Patrick et al. 1993). Combined, our results suggest that this may be due to the compromised integrity and atypical function of the somatosensory and insular loops of the emotion circuit that serve the generation of the bodily component of emotion. The aversive somatic markers generated by SI–SII and insula when seeing aggressive cues could inhibit aggressive behavior (Blair 2001). Thus their compromised function and structure may lead to disinhibited affect due to lacking somatic feedback from evoked emotions during affective provocation, potentially predisposing to aggression. Yet, it must be noted that some prior studies have found that psychopathic offenders show lower rather than greater neural responses during passive viewing of empathy-evoking scenarios (e.g., Meffert et al. 2013). We have no clear explanation for this, but it is possible that it pertains to the degree of aggressive violence present in the scenes (e.g., fights and assaults versus controlled minor pain inflicted on hands) or the general context of the aversive stimulation (complex and naturalistic versus well-controlled).

Criminal psychopathy was also associated with lowered connectivity of the key nodes of the social and emotional brain networks, including amygdala, insula, thalamus, and frontal pole. This effect was however specific to the incarcerated subjects and was not consistently replicated in the community sample as a function of psychopathic traits. Thus, even though there are parallels in the regional responsiveness of the brain’s affective circuit in the convicted psychopaths and well-functioning subjects with psychopathic traits, it is likely that the disrupted functional connectivity of this network is specific to criminal psychopathy.

### Limitations and Future Directions

The psychopathic traits of the community sample were assessed using self-reports (LSRP) rather than PCL-R, as the latter is based on interviews and extensive review of collateral information (Hare 1991). The nature of psychopathic personality per se (e.g., tendency to lie) makes self-report scales difficult to construct. Numerous studies have however confirmed that self-reports on psychopathic traits predict real-life antisocial behavior (Furnham et al. 2013); the brain imaging results from the forensic sample (evaluated with PCL-R) and community sample (evaluated with self-reports) were concordant. We aimed at recruiting prisoner subjects not using antipsychotics, antidepressants or anxiolytics; however, it was not possible to recruit a completely medication-free sample. The convicted offenders and healthy controls also differ from each other regarding the available quality and quantity of social interaction, leisure time activities and so forth. Ideally, this kind of study should thus also involve a forensic but non-psychopathic sample. Our data are also cross-sectional in nature and cannot resolve the potential causal link between the cerebral structural and functional alterations and psychopathic traits. Because conduct problems in adolescence are predictive of adult psychopathy (Burke et al. 2007), future longitudinal studies are needed to delineate the developmental neural pathways of psychopathy and assess the contribution of early social environment to the observed neural alterations.

Our naturalistic movie viewing paradigm yields a high-dimensional stimulus space and it is difficult to tease apart the specific contribution of different aspects of violence (such as facial expressions and vocalizations). Yet, such isolated features are not representative of the real world either (Adolphs et al. 2016). Because evolution has prepared the brain to process a continuous sensory stream rather than “snapshots” such as pictures and sound bursts, dynamic natural stimuli trigger more consistent and stronger neural responses than the conventionally used well-controlled yet reduced stimuli (Yao et al. 2007; Fox et al. 2009; Schultz et al. 2013). Violent episodes span numerous overlapping and hierarchic time scales necessitating parallel processing of multiple sensory features. Consequently, they cannot be adequately explored with fully controlled classic experimental designs.

## Conclusions

Normally functioning individuals with high psychopathic traits have structural and functional brain characteristics that are similar to violent offender with high psychopathic traits. These characteristics include both fronto-limbic cortical atrophy and enhanced brain activity in affective circuits while seeing violence. Altogether these data show that integrity and function of the frontal and insular cortex associate with both extreme and benign variations of antisocial behavior, providing neurobiological support for a common neural basis of antisocial behaviors with different severity.

## Supplementary Material

Nummenmaa_et_al_SI-R1_bhab072

## Notes

We acknowledge director Juhani Järvi and other staff members of Turku Prison who made it possible to safely transport and guard the inmates during the neuroimaging. *Conflict of Interest:* The authors declare no conflict of interest.
