# Supplementary material for: Brain Basis of Psychopathy in Criminal Offenders and General Population
Source: Cereb Cortex. 2021 Apr 9;31(9):4104–14. doi: 10.1093/cercor/bhab072 (PMC8328218; doi:10.1093/cercor/bhab072)
Supplement: Nummenmaa_et_al_SI-R1_bhab072 [file nummenmaa_et_al_si-r1_bhab072.docx]

**Table S-1.** Medications taken by the prisoner subjects. Note that antidepressant, anxiolytics and antipsychotics were terminated two weeks before the study.

| **Medication type** | **Number of subjects taking medication** | **List of medicines** |
| --- | --- | --- |
| Antidepressants | 3 | Citalopram, Fluoxetine, Venlafaxine |
| Anxiolytics | 5 | Buspirone, Hydroxyzine, Propranolol |
| Antipsychotics | 1 | Risperidone |
| Insomnia medication | 14 | Amitriptyline,Hydroxyzine, Melatonin, Mirtazapine, Quetiapine |
| Thyroid medication | 1 | Levothyroxine |
| Other stimulants | 1 | Atomoxetine |

**Table S-2**. Psychiatric diagnoses of the prisoner subjects

| **Diagnosis** | **ICD code** | **Number of subjects** |
| --- | --- | --- |
| Moderate depressive episode | F32.10 | 1 |
| Dysthymia | F34.1 | 1 |
| Unspecified anxiety disorder | F41.09 | 1 |
| Other mixed anxiety disorders | F41.3 | 1 |
| Adjustment disorder | F43.22 | 1 |
| Asocial personality disorder | F60.2 | 4 |
| Emotionally unstable personality disorder | F60.3 | 1 |
| Mixed and other personality disorders | F61.0 | 1 |
| Developmental disorder of scholastic skills, unspecified | F81.9 | 1 |
| Disturbance of activity and attention | F90 | 2 |


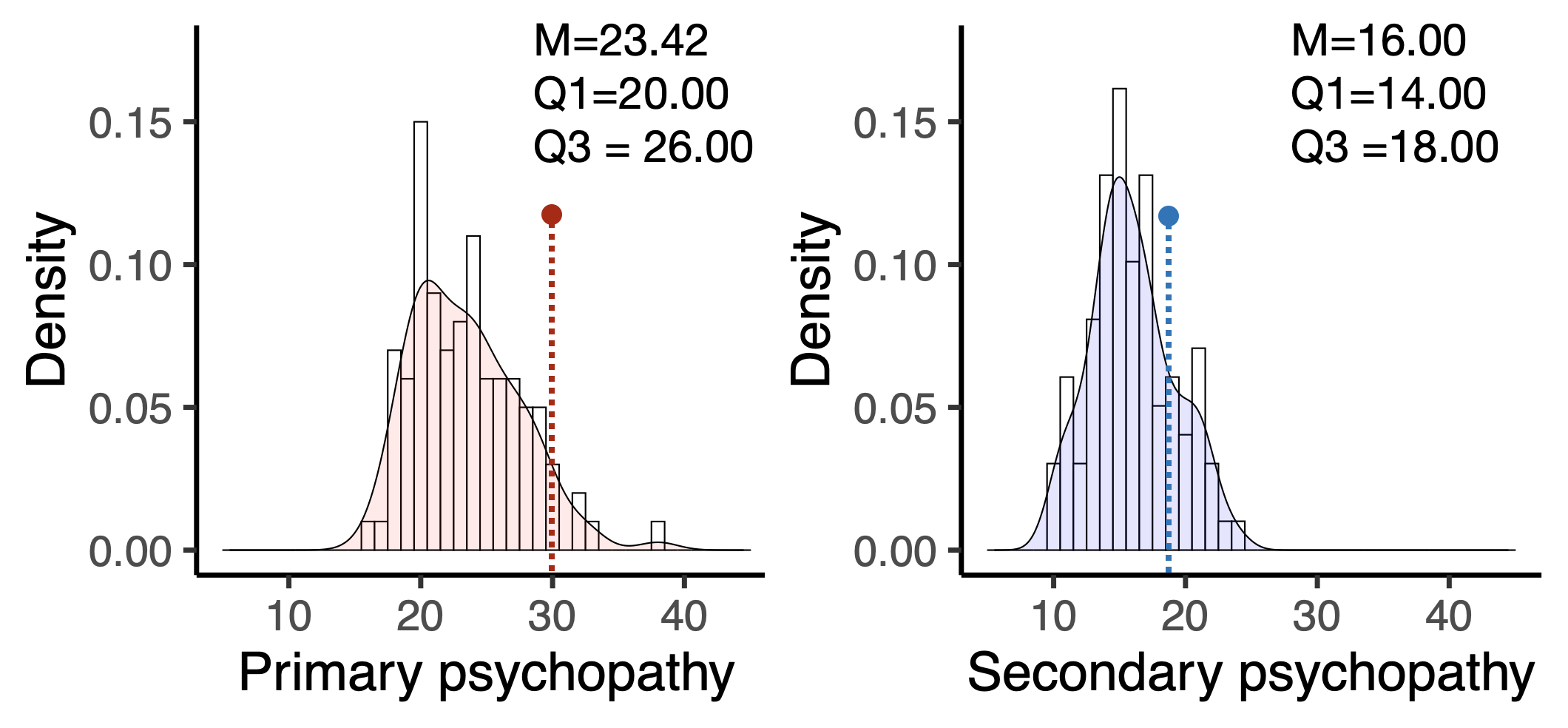


**Figure S-1**. Distribution of the primary and secondary psychopathy scores in the community sample. Dashed lines show mean LSRP scores from 10 offenders from whom these were available. Although these are clearly higher than the means of the community sample (30.1 vs 23.42 for primary and 19.3 vs. 15.99 for secondary psychopathy), these should be interpreted with caution as self-report scores are deemed unreliable in criminal psychopathy.
